# CORRIGENDUM

**DOI:** 10.1111/1759-7714.14748

**Published:** 2022-12-14

In Linfang Wu *et a*l.^1^ the following error was published on page 1588.

The authors noticed that the legends were wrong for Figure 3d. In Figure 3d, the non‐G3 RILT group should have superior overall survival and its K‐M curve should be on the upper side of the G3 RILT group. The correct image is shown below:

**FIGURE 3:**
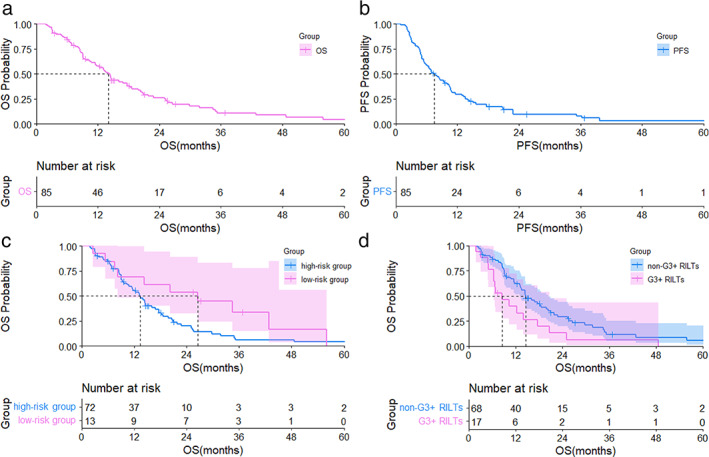
(a) Overall survival curve for patients with stage III NSCLC and ILD treated with TRT; (b) PFS curve for patients with stage III NSCLC andILD treated with thoracic radiation therapy; (c) overall survival curve for 13 patients in the low‐risk group and 72 patients in the high‐risk group; (d) overall survival curve for 17 patients with G3+ RILTs and 68 patients without G3 RILTs. OS, overall survival; PFS, progression‐free survival; low‐risk group, the patients with V20 < 20% and honeycombing score <1; NSCLC, non‐small‐cell lung cancer; ILD, interstitial lung disease; RILTs, radiation‐induced lung toxicities; CI, confidence interval; HR, hazard ratio

The authors apologize for the error and any inconvenience it may have caused.
